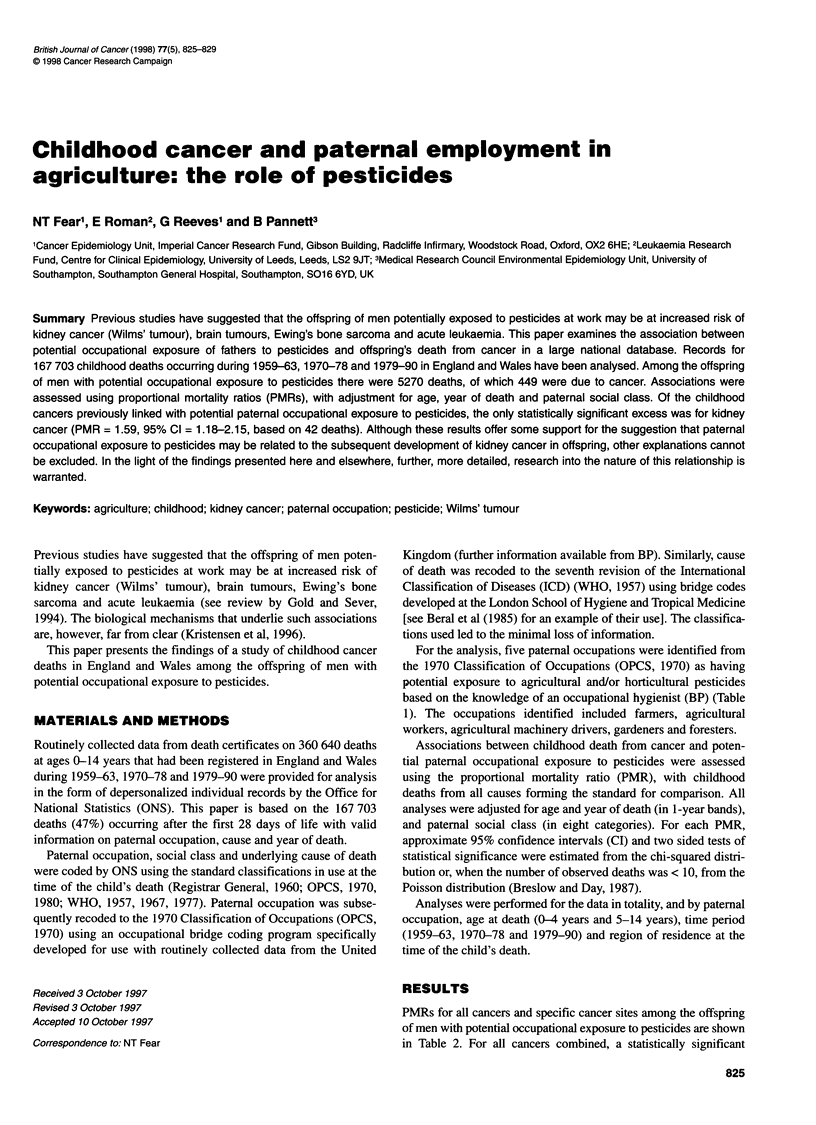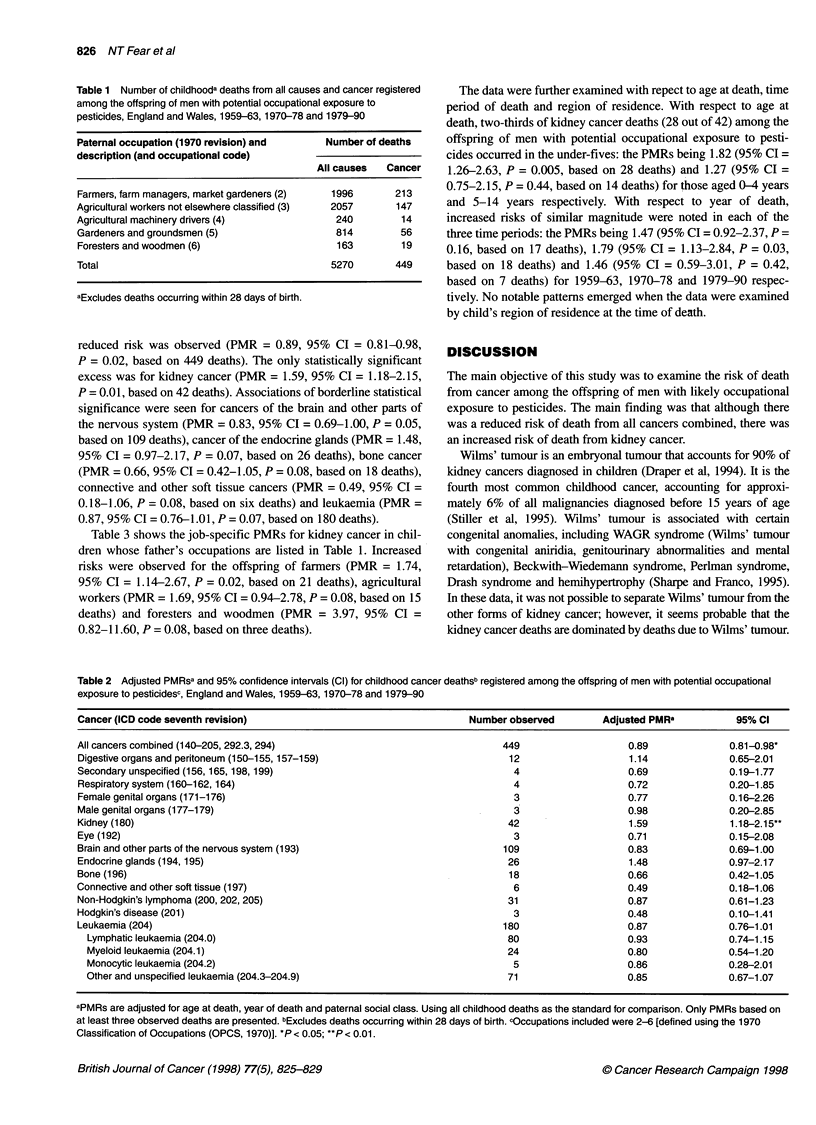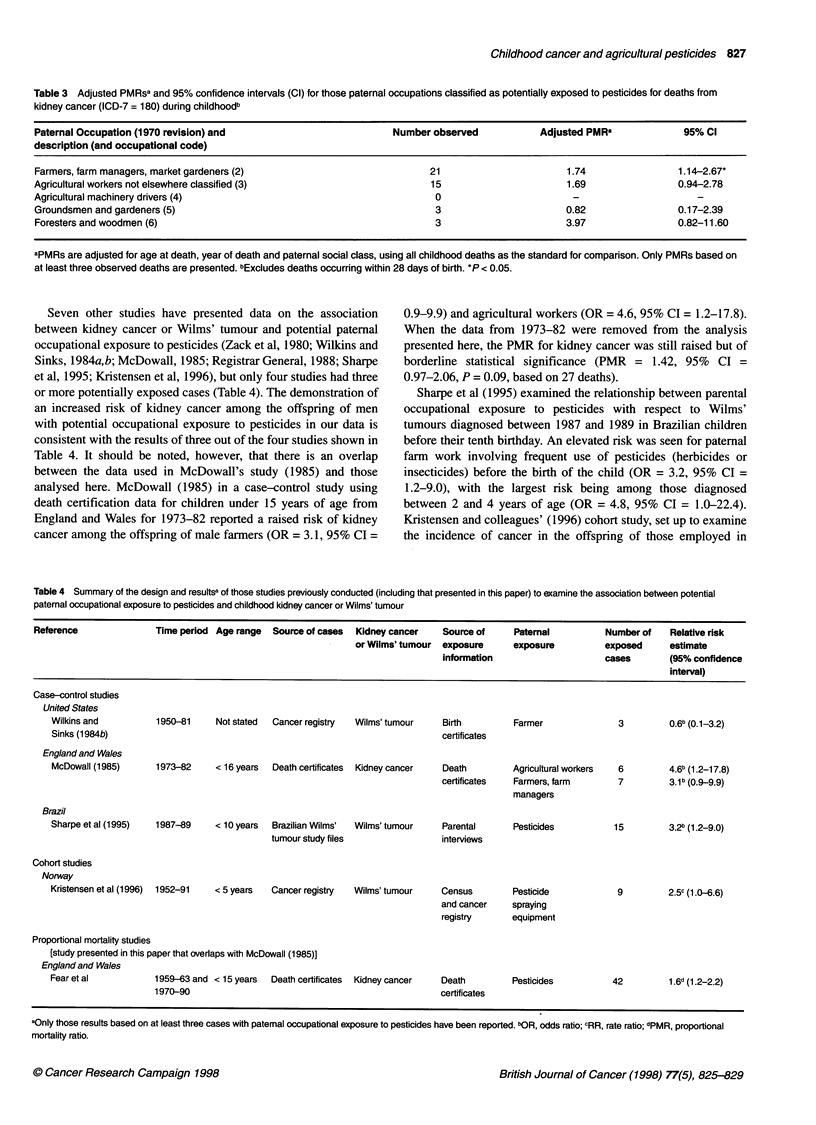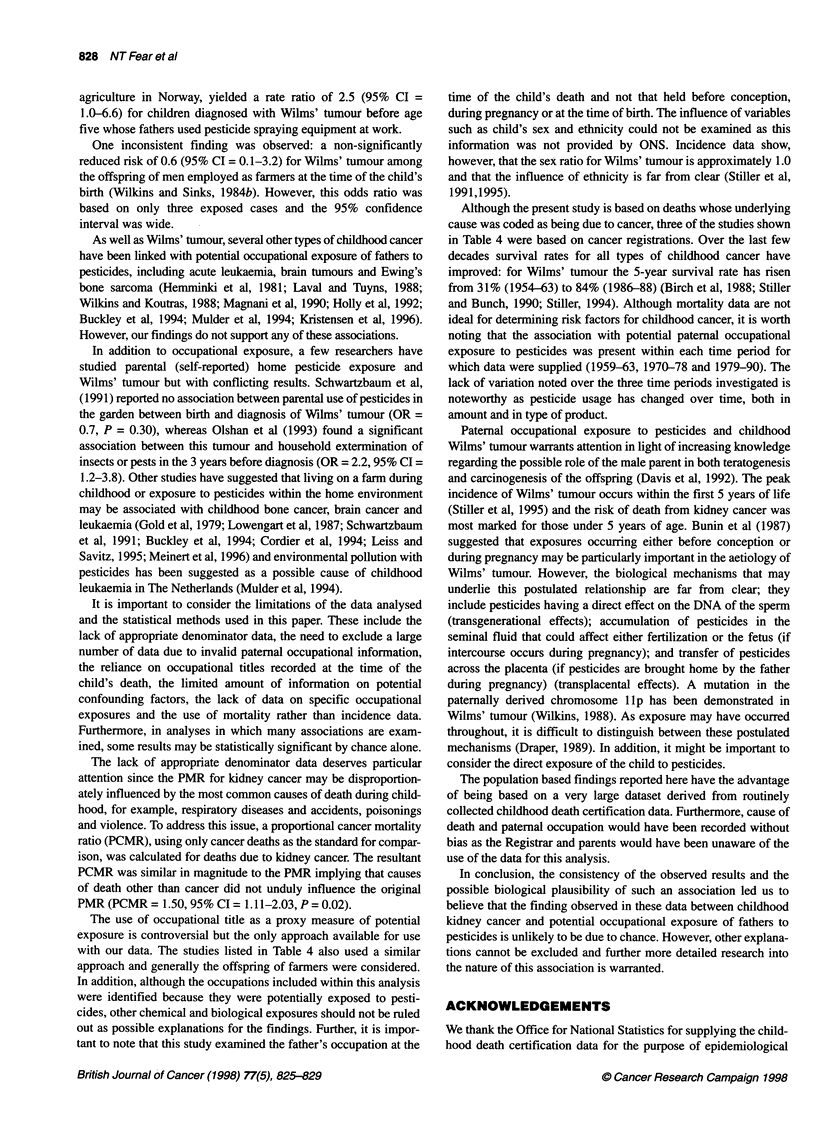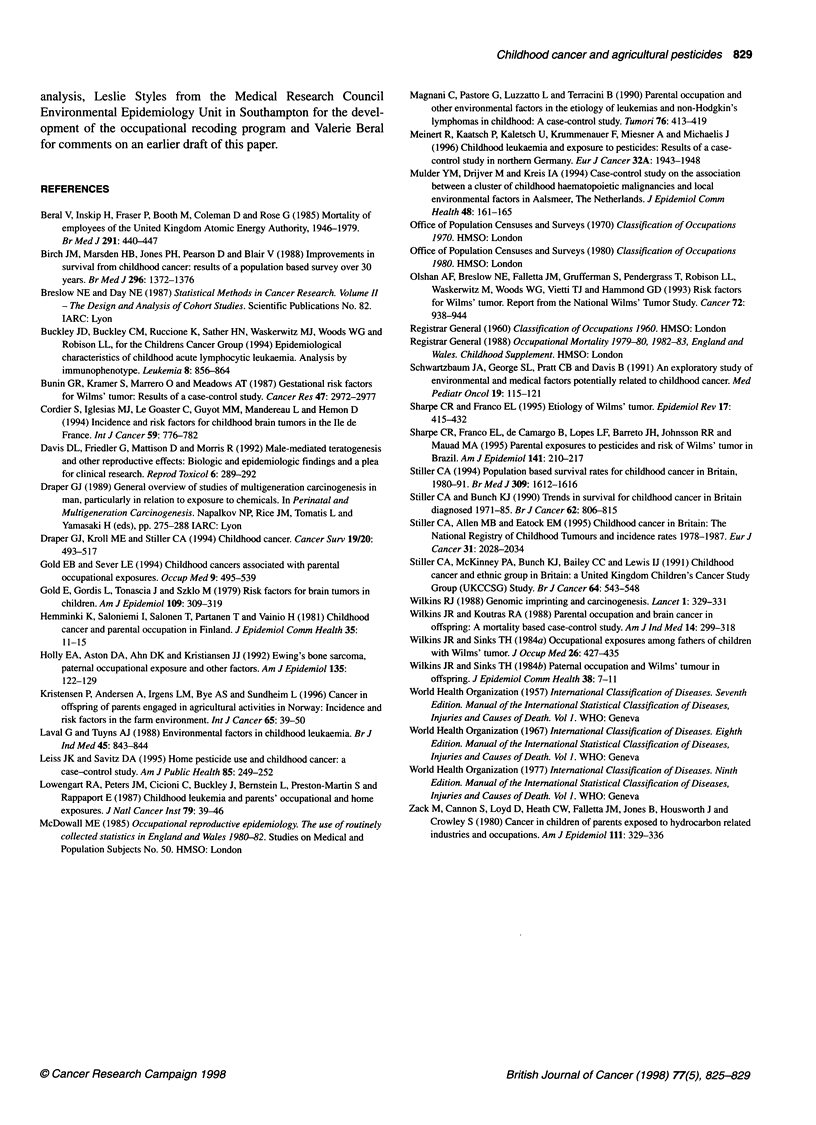# Childhood cancer and paternal employment in agriculture: the role of pesticides.

**DOI:** 10.1038/bjc.1998.134

**Published:** 1998-03

**Authors:** N. T. Fear, E. Roman, G. Reeves, B. Pannett

**Affiliations:** Cancer Epidemiology Unit, Imperial Cancer Research Fund, Radcliffe Infirmary, Oxford, UK.

## Abstract

Previous studies have suggested that the offspring of men potentially exposed to pesticides at work may be at increased risk of kidney cancer (Wilms' tumour), brain tumours, Ewing's bone sarcoma and acute leukaemia. This paper examines the association between potential occupational exposure of fathers to pesticides and offspring's death from cancer in a large national database. Records for 167703 childhood deaths occurring during 1959-63, 1970-78 and 1979-90 in England and Wales have been analysed. Among the offspring of men with potential occupational exposure to pesticides there were 5270 deaths, of which 449 were due to cancer. Associations were assessed using proportional mortality ratios (PMRs), with adjustment for age, year of death and paternal social class. Of the childhood cancers previously linked with potential paternal occupational exposure to pesticides, the only statistically significant excess was for kidney cancer (PMR=1.59, 95% CI=1.18-2.15, based on 42 deaths). Although these results offer some support for the suggestion that paternal occupational exposure to pesticides may be related to the subsequent development of kidney cancer in offspring, other explanations cannot be excluded. In the light of the findings presented here and elsewhere, further, more detailed, research into the nature of this relationship is warranted.


British Journal of Cancer (1998) 77(5), 825–829
© 1998 Cancer Research Campaign

### Childhood cancer and paternal employment in agriculture: the role of pesticides

**NT Fear[1], E Roman[2], G Reeves[1] and B Pannett[3]**

[1]Cancer Epidemiology Unit, Imperial Cancer Research Fund, Gibson Building, Radcliffe Infirmary, Woodstock Road, Oxford, OX2 6HE; [2]Leukaemia Research Fund, Centre for Clinical Epidemiology, University of Leeds, Leeds, LS2 9JT; [3]Medical Research Council Environmental Epidemiology Unit, University of Southampton, Southampton General Hospital, Southampton, SO16 6YD, UK

**Summary** Previous studies have suggested that the offspring of men potentially exposed to pesticides at work may be at increased risk of kidney cancer (Wilms' tumour), brain tumours, Ewing's bone sarcoma and acute leukaemia. This paper examines the association between potential occupational exposure of fathers to pesticides and offspring's death from cancer in a large national database. Records for 167 703 childhood deaths occurring during 1959–63, 1970–78 and 1979–90 in England and Wales have been analysed. Among the offspring of men with potential occupational exposure to pesticides there were 5270 deaths, of which 449 were due to cancer. Associations were assessed using proportional mortality ratios (PMRs), with adjustment for age, year of death and paternal social class. Of the childhood cancers previously linked with potential paternal occupational exposure to pesticides, the only statistically significant excess was for kidney cancer (PMR = 1.59, 95% CI = 1.18–2.15, based on 42 deaths). Although these results offer some support for the suggestion that paternal occupational exposure to pesticides may be related to the subsequent development of kidney cancer in offspring, other explanations cannot be excluded. In the light of the findings presented here and elsewhere, further, more detailed, research into the nature of this relationship is warranted.

**Keywords:** agriculture; childhood; kidney cancer; paternal occupation; pesticide; Wilms' tumour

Previous studies have suggested that the offspring of men potentially exposed to pesticides at work may be at increased risk of kidney cancer (Wilms' tumour), brain tumours, Ewing's bone sarcoma and acute leukaemia (see review by Gold and Sever, 1994). The biological mechanisms that underlie such associations are, however, far from clear (Kristensen et al, 1996).

This paper presents the findings of a study of childhood cancer deaths in England and Wales among the offspring of men with potential occupational exposure to pesticides.

### MATERIALS AND METHODS

Routinely collected data from death certificates on 360 640 deaths at ages 0–14 years that had been registered in England and Wales during 1959–63, 1970–78 and 1979–90 were provided for analysis in the form of depersonalized individual records by the Office for National Statistics (ONS). This paper is based on the 167 703 deaths (47%) occurring after the first 28 days of life with valid information on paternal occupation, cause and year of death.

Paternal occupation, social class and underlying cause of death were coded by ONS using the standard classifications in use at the time of the child's death (Registrar General, 1960; OPCS, 1970, 1980; WHO, 1957, 1967, 1977). Paternal occupation was subsequently recoded to the 1970 Classification of Occupations (OPCS, 1970) using an occupational bridge coding program specifically developed for use with routinely collected data from the United Kingdom (further information available from BP). Similarly, cause of death was recoded to the seventh revision of the International Classification of Diseases (ICD) (WHO, 1957) using bridge codes developed at the London School of Hygiene and Tropical Medicine [see Beral et al (1985) for an example of their use]. The classifications used led to the minimal loss of information.

For the analysis, five paternal occupations were identified from the 1970 Classification of Occupations (OPCS, 1970) as having potential exposure to agricultural and/or horticultural pesticides based on the knowledge of an occupational hygienist (BP) (Table 1). The occupations identified included farmers, agricultural workers, agricultural machinery drivers, gardeners and foresters.

Associations between childhood death from cancer and potential paternal occupational exposure to pesticides were assessed using the proportional mortality ratio (PMR), with childhood deaths from all causes forming the standard for comparison. All analyses were adjusted for age and year of death (in 1-year bands), and paternal social class (in eight categories). For each PMR, approximate 95% confidence intervals (CI) and two sided tests of statistical significance were estimated from the chi-squared distribution or, when the number of observed deaths was < 10, from the Poisson distribution (Breslow and Day, 1987).

Analyses were performed for the data in totality, and by paternal occupation, age at death (0–4 years and 5–14 years), time period (1959–63, 1970–78 and 1979–90) and region of residence at the time of the child's death.

### RESULTS

PMRs for all cancers and specific cancer sites among the offspring of men with potential occupational exposure to pesticides are shown in Table 2. For all cancers combined, a statistically significant

*Received 3 October 1997*
*Revised 3 October 1997*
*Accepted 10 October 1997*

*Correspondence to:* NT Fear

Table 1 Number of childhood[a] deaths from all causes and cancer registered among the offspring of men with potential occupational exposure to pesticides, England and Wales, 1959–63, 1970–78 and 1979–90

| Paternal occupation (1970 revision) and description (and occupational code) | Number of deaths | |
|---|---|---|
| | All causes | Cancer |
| Farmers, farm managers, market gardeners (2) | 1996 | 213 |
| Agricultural workers not elsewhere classified (3) | 2057 | 147 |
| Agricultural machinery drivers (4) | 240 | 14 |
| Gardeners and groundsmen (5) | 814 | 56 |
| Foresters and woodmen (6) | 163 | 19 |
| Total | 5270 | 449 |

[a]Excludes deaths occurring within 28 days of birth.

reduced risk was observed (PMR = 0.89, 95% CI = 0.81–0.98, $P$ = 0.02, based on 449 deaths). The only statistically significant excess was for kidney cancer (PMR = 1.59, 95% CI = 1.18–2.15, $P$ = 0.01, based on 42 deaths). Associations of borderline statistical significance were seen for cancers of the brain and other parts of the nervous system (PMR = 0.83, 95% CI = 0.69–1.00, $P$ = 0.05, based on 109 deaths), cancer of the endocrine glands (PMR = 1.48, 95% CI = 0.97–2.17, $P$ = 0.07, based on 26 deaths), bone cancer (PMR = 0.66, 95% CI = 0.42–1.05, $P$ = 0.08, based on 18 deaths), connective and other soft tissue cancers (PMR = 0.49, 95% CI = 0.18–1.06, $P$ = 0.08, based on six deaths) and leukaemia (PMR = 0.87, 95% CI = 0.76–1.01, $P$ = 0.07, based on 180 deaths).

Table 3 shows the job-specific PMRs for kidney cancer in children whose father's occupations are listed in Table 1. Increased risks were observed for the offspring of farmers (PMR = 1.74, 95% CI = 1.14–2.67, $P$ = 0.02, based on 21 deaths), agricultural workers (PMR = 1.69, 95% CI = 0.94–2.78, $P$ = 0.08, based on 15 deaths) and foresters and woodmen (PMR = 3.97, 95% CI = 0.82–11.60, $P$ = 0.08, based on three deaths).

The data were further examined with repect to age at death, time period of death and region of residence. With respect to age at death, two-thirds of kidney cancer deaths (28 out of 42) among the offspring of men with potential occupational exposure to pesticides occurred in the under-fives: the PMRs being 1.82 (95% CI = 1.26–2.63, $P$ = 0.005, based on 28 deaths) and 1.27 (95% CI = 0.75–2.15, $P$ = 0.44, based on 14 deaths) for those aged 0–4 years and 5–14 years respectively. With respect to year of death, increased risks of similar magnitude were noted in each of the three time periods: the PMRs being 1.47 (95% CI = 0.92–2.37, $P$ = 0.16, based on 17 deaths), 1.79 (95% CI = 1.13–2.84, $P$ = 0.03, based on 18 deaths) and 1.46 (95% CI = 0.59–3.01, $P$ = 0.42, based on 7 deaths) for 1959–63, 1970–78 and 1979–90 respectively. No notable patterns emerged when the data were examined by child's region of residence at the time of death.

### DISCUSSION

The main objective of this study was to examine the risk of death from cancer among the offspring of men with likely occupational exposure to pesticides. The main finding was that although there was a reduced risk of death from all cancers combined, there was an increased risk of death from kidney cancer.

Wilms' tumour is an embryonal tumour that accounts for 90% of kidney cancers diagnosed in children (Draper et al, 1994). It is the fourth most common childhood cancer, accounting for approximately 6% of all malignancies diagnosed before 15 years of age (Stiller et al, 1995). Wilms' tumour is associated with certain congenital anomalies, including WAGR syndrome (Wilms' tumour with congenital aniridia, genitourinary abnormalities and mental retardation), Beckwith–Wiedemann syndrome, Perlman syndrome, Drash syndrome and hemihypertrophy (Sharpe and Franco, 1995). In these data, it was not possible to separate Wilms' tumour from the other forms of kidney cancer; however, it seems probable that the kidney cancer deaths are dominated by deaths due to Wilms' tumour.

Table 2 Adjusted PMRs[a] and 95% confidence intervals (CI) for childhood cancer deaths[b] registered among the offspring of men with potential occupational exposure to pesticides[c], England and Wales, 1959–63, 1970–78 and 1979–90

| Cancer (ICD code seventh revision) | Number observed | Adjusted PMR[a] | 95% CI |
|---|---|---|---|
| All cancers combined (140–205, 292.3, 294) | 449 | 0.89 | 0.81–0.98* |
| Digestive organs and peritoneum (150–155, 157–159) | 12 | 1.14 | 0.65–2.01 |
| Secondary unspecified (156, 165, 198, 199) | 4 | 0.69 | 0.19–1.77 |
| Respiratory system (160–162, 164) | 4 | 0.72 | 0.20–1.85 |
| Female genital organs (171–176) | 3 | 0.77 | 0.16–2.26 |
| Male genital organs (177–179) | 3 | 0.98 | 0.20–2.85 |
| Kidney (180) | 42 | 1.59 | 1.18–2.15** |
| Eye (192) | 3 | 0.71 | 0.15–2.08 |
| Brain and other parts of the nervous system (193) | 109 | 0.83 | 0.69–1.00 |
| Endocrine glands (194, 195) | 26 | 1.48 | 0.97–2.17 |
| Bone (196) | 18 | 0.66 | 0.42–1.05 |
| Connective and other soft tissue (197) | 6 | 0.49 | 0.18–1.06 |
| Non-Hodgkin's lymphoma (200, 202, 205) | 31 | 0.87 | 0.61–1.23 |
| Hodgkin's disease (201) | 3 | 0.48 | 0.10–1.41 |
| Leukaemia (204) | 180 | 0.87 | 0.76–1.01 |
| Lymphatic leukaemia (204.0) | 80 | 0.93 | 0.74–1.15 |
| Myeloid leukaemia (204.1) | 24 | 0.80 | 0.54–1.20 |
| Monocytic leukaemia (204.2) | 5 | 0.86 | 0.28–2.01 |
| Other and unspecified leukaemia (204.3–204.9) | 71 | 0.85 | 0.67–1.07 |

[a]PMRs are adjusted for age at death, year of death and paternal social class. Using all childhood deaths as the standard for comparison. Only PMRs based on at least three observed deaths are presented. [b]Excludes deaths occurring within 28 days of birth. [c]Occupations included were 2–6 [defined using the 1970 Classification of Occupations (OPCS, 1970)]. $^*P < 0.05$; $^{**}P < 0.01$.

© Cancer Research Campaign 1998

**Table 3** Adjusted PMRs[a] and 95% confidence intervals (CI) for those paternal occupations classified as potentially exposed to pesticides for deaths from kidney cancer (ICD-7 = 180) during childhood[b]

| Paternal Occupation (1970 revision) and description (and occupational code) | Number observed | Adjusted PMR[a] | 95% CI |
|---|---|---|---|
| Farmers, farm managers, market gardeners (2) | 21 | 1.74 | 1.14–2.67* |
| Agricultural workers not elsewhere classified (3) | 15 | 1.69 | 0.94–2.78 |
| Agricultural machinery drivers (4) | 0 | – | – |
| Groundsmen and gardeners (5) | 3 | 0.82 | 0.17–2.39 |
| Foresters and woodmen (6) | 3 | 3.97 | 0.82–11.60 |

[a]PMRs are adjusted for age at death, year of death and paternal social class, using all childhood deaths as the standard for comparison. Only PMRs based on at least three observed deaths are presented. [b]Excludes deaths occurring within 28 days of birth. *$P < 0.05$.

Seven other studies have presented data on the association between kidney cancer or Wilms' tumour and potential paternal occupational exposure to pesticides (Zack et al, 1980; Wilkins and Sinks, 1984*a*,*b*; McDowall, 1985; Registrar General, 1988; Sharpe et al, 1995; Kristensen et al, 1996), but only four studies had three or more potentially exposed cases (Table 4). The demonstration of an increased risk of kidney cancer among the offspring of men with potential occupational exposure to pesticides in our data is consistent with the results of three out of the four studies shown in Table 4. It should be noted, however, that there is an overlap between the data used in McDowall's study (1985) and those analysed here. McDowall (1985) in a case–control study using death certification data for children under 15 years of age from England and Wales for 1973–82 reported a raised risk of kidney cancer among the offspring of male farmers (OR = 3.1, 95% CI = 0.9–9.9) and agricultural workers (OR = 4.6, 95% CI = 1.2–17.8). When the data from 1973–82 were removed from the analysis presented here, the PMR for kidney cancer was still raised but of borderline statistical significance (PMR = 1.42, 95% CI = 0.97–2.06, $P = 0.09$, based on 27 deaths).

Sharpe et al (1995) examined the relationship between parental occupational exposure to pesticides with respect to Wilms' tumours diagnosed between 1987 and 1989 in Brazilian children before their tenth birthday. An elevated risk was seen for paternal farm work involving frequent use of pesticides (herbicides or insecticides) before the birth of the child (OR = 3.2, 95% CI = 1.2–9.0), with the largest risk being among those diagnosed between 2 and 4 years of age (OR = 4.8, 95% CI = 1.0–22.4). Kristensen and colleagues' (1996) cohort study, set up to examine the incidence of cancer in the offspring of those employed in

**Table 4** Summary of the design and results[a] of those studies previously conducted (including that presented in this paper) to examine the association between potential paternal occupational exposure to pesticides and childhood kidney cancer or Wilms' tumour

| Reference | Time period | Age range | Source of cases | Kidney cancer or Wilms' tumour | Source of exposure information | Paternal exposure | Number of exposed cases | Relative risk estimate (95% confidence interval) |
|---|---|---|---|---|---|---|---|---|
| Case–control studies | | | | | | | | |
| *United States* | | | | | | | | |
| Wilkins and Sinks (1984*b*) | 1950–81 | Not stated | Cancer registry | Wilms' tumour | Birth certificates | Farmer | 3 | 0.6[b] (0.1–3.2) |
| *England and Wales* | | | | | | | | |
| McDowall (1985) | 1973–82 | < 16 years | Death certificates | Kidney cancer | Death certificates | Agricultural workers | 6 | 4.6[b] (1.2–17.8) |
| | | | | | | Farmers, farm managers | 7 | 3.1[b] (0.9–9.9) |
| *Brazil* | | | | | | | | |
| Sharpe et al (1995) | 1987–89 | < 10 years | Brazilian Wilms' tumour study files | Wilms' tumour | Parental interviews | Pesticides | 15 | 3.2[b] (1.2–9.0) |
| Cohort studies | | | | | | | | |
| *Norway* | | | | | | | | |
| Kristensen et al (1996) | 1952–91 | < 5 years | Cancer registry | Wilms' tumour | Census and cancer registry | Pesticide spraying equipment | 9 | 2.5[c] (1.0–6.6) |
| Proportional mortality studies [study presented in this paper that overlaps with McDowall (1985)] | | | | | | | | |
| *England and Wales* | | | | | | | | |
| Fear et al | 1959–63 and 1970–90 | < 15 years | Death certificates | Kidney cancer | Death certificates | Pesticides | 42 | 1.6[d] (1.2–2.2) |

[a]Only those results based on at least three cases with paternal occupational exposure to pesticides have been reported. [b]OR, odds ratio; [c]RR, rate ratio; [d]PMR, proportional mortality ratio.

© Cancer Research Campaign 1998

agriculture in Norway, yielded a rate ratio of 2.5 (95% CI = 1.0–6.6) for children diagnosed with Wilms' tumour before age five whose fathers used pesticide spraying equipment at work.

One inconsistent finding was observed: a non-significantly reduced risk of 0.6 (95% CI = 0.1–3.2) for Wilms' tumour among the offspring of men employed as farmers at the time of the child's birth (Wilkins and Sinks, 1984*b*). However, this odds ratio was based on only three exposed cases and the 95% confidence interval was wide.

As well as Wilms' tumour, several other types of childhood cancer have been linked with potential occupational exposure of fathers to pesticides, including acute leukaemia, brain tumours and Ewing's bone sarcoma (Hemminki et al, 1981; Laval and Tuyns, 1988; Wilkins and Koutras, 1988; Magnani et al, 1990; Holly et al, 1992; Buckley et al, 1994; Mulder et al, 1994; Kristensen et al, 1996). However, our findings do not support any of these associations.

In addition to occupational exposure, a few researchers have studied parental (self-reported) home pesticide exposure and Wilms' tumour but with conflicting results. Schwartzbaum et al, (1991) reported no association between parental use of pesticides in the garden between birth and diagnosis of Wilms' tumour (OR = 0.7, $P = 0.30$), whereas Olshan et al (1993) found a significant association between this tumour and household extermination of insects or pests in the 3 years before diagnosis (OR = 2.2, 95% CI = 1.2–3.8). Other studies have suggested that living on a farm during childhood or exposure to pesticides within the home environment may be associated with childhood bone cancer, brain cancer and leukaemia (Gold et al, 1979; Lowengart et al, 1987; Schwartzbaum et al, 1991; Buckley et al, 1994; Cordier et al, 1994; Leiss and Savitz, 1995; Meinert et al, 1996) and environmental pollution with pesticides has been suggested as a possible cause of childhood leukaemia in The Netherlands (Mulder et al, 1994).

It is important to consider the limitations of the data analysed and the statistical methods used in this paper. These include the lack of appropriate denominator data, the need to exclude a large number of data due to invalid paternal occupational information, the reliance on occupational titles recorded at the time of the child's death, the limited amount of information on potential confounding factors, the lack of data on specific occupational exposures and the use of mortality rather than incidence data. Furthermore, in analyses in which many associations are examined, some results may be statistically significant by chance alone.

The lack of appropriate denominator data deserves particular attention since the PMR for kidney cancer may be disproportionately influenced by the most common causes of death during childhood, for example, respiratory diseases and accidents, poisonings and violence. To address this issue, a proportional cancer mortality ratio (PCMR), using only cancer deaths as the standard for comparison, was calculated for deaths due to kidney cancer. The resultant PCMR was similar in magnitude to the PMR implying that causes of death other than cancer did not unduly influence the original PMR (PCMR = 1.50, 95% CI = 1.11–2.03, $P = 0.02$).

The use of occupational title as a proxy measure of potential exposure is controversial but the only approach available for use with our data. The studies listed in Table 4 also used a similar approach and generally the offspring of farmers were considered. In addition, although the occupations included within this analysis were identified because they were potentially exposed to pesticides, other chemical and biological exposures should not be ruled out as possible explanations for the findings. Further, it is important to note that this study examined the father's occupation at the time of the child's death and not that held before conception, during pregnancy or at the time of birth. The influence of variables such as child's sex and ethnicity could not be examined as this information was not provided by ONS. Incidence data show, however, that the sex ratio for Wilms' tumour is approximately 1.0 and that the influence of ethnicity is far from clear (Stiller et al, 1991,1995).

Although the present study is based on deaths whose underlying cause was coded as being due to cancer, three of the studies shown in Table 4 were based on cancer registrations. Over the last few decades survival rates for all types of childhood cancer have improved: for Wilms' tumour the 5-year survival rate has risen from 31% (1954–63) to 84% (1986–88) (Birch et al, 1988; Stiller and Bunch, 1990; Stiller, 1994). Although mortality data are not ideal for determining risk factors for childhood cancer, it is worth noting that the association with potential paternal occupational exposure to pesticides was present within each time period for which data were supplied (1959–63, 1970–78 and 1979–90). The lack of variation noted over the three time periods investigated is noteworthy as pesticide usage has changed over time, both in amount and in type of product.

Paternal occupational exposure to pesticides and childhood Wilms' tumour warrants attention in light of increasing knowledge regarding the possible role of the male parent in both teratogenesis and carcinogenesis of the offspring (Davis et al, 1992). The peak incidence of Wilms' tumour occurs within the first 5 years of life (Stiller et al, 1995) and the risk of death from kidney cancer was most marked for those under 5 years of age. Bunin et al (1987) suggested that exposures occurring either before conception or during pregnancy may be particularly important in the aetiology of Wilms' tumour. However, the biological mechanisms that may underlie this postulated relationship are far from clear; they include pesticides having a direct effect on the DNA of the sperm (transgenerational effects); accumulation of pesticides in the seminal fluid that could affect either fertilization or the fetus (if intercourse occurs during pregnancy); and transfer of pesticides across the placenta (if pesticides are brought home by the father during pregnancy) (transplacental effects). A mutation in the paternally derived chromosome 11p has been demonstrated in Wilms' tumour (Wilkins, 1988). As exposure may have occurred throughout, it is difficult to distinguish between these postulated mechanisms (Draper, 1989). In addition, it might be important to consider the direct exposure of the child to pesticides.

The population based findings reported here have the advantage of being based on a very large dataset derived from routinely collected childhood death certification data. Furthermore, cause of death and paternal occupation would have been recorded without bias as the Registrar and parents would have been unaware of the use of the data for this analysis.

In conclusion, the consistency of the observed results and the possible biological plausibility of such an association led us to believe that the finding observed in these data between childhood kidney cancer and potential occupational exposure of fathers to pesticides is unlikely to be due to chance. However, other explanations cannot be excluded and further more detailed research into the nature of this association is warranted.

### ACKNOWLEDGEMENTS

We thank the Office for National Statistics for supplying the childhood death certification data for the purpose of epidemiological

© Cancer Research Campaign 1998

analysis, Leslie Styles from the Medical Research Council Environmental Epidemiology Unit in Southampton for the development of the occupational recoding program and Valerie Beral for comments on an earlier draft of this paper.

### REFERENCES

Beral V, Inskip H, Fraser P, Booth M, Coleman D and Rose G (1985) Mortality of employees of the United Kingdom Atomic Energy Authority, 1946–1979. *Br Med J* **291**: 440–447

Birch JM, Marsden HB, Jones PH, Pearson D and Blair V (1988) Improvements in survival from childhood cancer: results of a population based survey over 30 years. *Br Med J* **296**: 1372–1376

Breslow NE and Day NE (1987) *Statistical Methods in Cancer Research. Volume II – The Design and Analysis of Cohort Studies*. Scientific Publications No. 82. IARC: Lyon

Buckley JD, Buckley CM, Ruccione K, Sather HN, Waskerwitz MJ, Woods WG and Robison LL, for the Childrens Cancer Group (1994) Epidemiological characteristics of childhood acute lymphocytic leukaemia. Analysis by immunophenotype. *Leukemia* **8**: 856–864

Bunin GR, Kramer S, Marrero O and Meadows AT (1987) Gestational risk factors for Wilms' tumor: Results of a case-control study. *Cancer Res* **47**: 2972–2977

Cordier S, Iglesias MJ, Le Goaster C, Guyot MM, Mandereau L and Hemon D (1994) Incidence and risk factors for childhood brain tumors in the Ile de France. *Int J Cancer* **59**: 776–782

Davis DL, Friedler G, Mattison D and Morris R (1992) Male-mediated teratogenesis and other reproductive effects: Biologic and epidemiologic findings and a plea for clinical research. *Reprod Toxicol* **6**: 289–292

Draper GJ (1989) General overview of studies of multigeneration carcinogenesis in man, particularly in relation to exposure to chemicals. In *Perinatal and Multigeneration Carcinogenesis*. Napalkov NP, Rice JM, Tomatis L and Yamasaki H (eds), pp. 275–288 IARC: Lyon

Draper GJ, Kroll ME and Stiller CA (1994) Childhood cancer. *Cancer Surv* **19/20**: 493–517

Gold EB and Sever LE (1994) Childhood cancers associated with parental occupational exposures. *Occup Med* **9**: 495–539

Gold E, Gordis L, Tonascia J and Szklo M (1979) Risk factors for brain tumors in children. *Am J Epidemiol* **109**: 309–319

Hemminki K, Saloniemi I, Salonen T, Partanen T and Vainio H (1981) Childhood cancer and parental occupation in Finland. *J Epidemiol Comm Health* **35**: 11–15

Holly EA, Aston DA, Ahn DK and Kristiansen JJ (1992) Ewing's bone sarcoma, paternal occupational exposure and other factors. *Am J Epidemiol* **135**: 122–129

Kristensen P, Andersen A, Irgens LM, Bye AS and Sundheim L (1996) Cancer in offspring of parents engaged in agricultural activities in Norway: Incidence and risk factors in the farm environment. *Int J Cancer* **65**: 39–50

Laval G and Tuyns AJ (1988) Environmental factors in childhood leukaemia. *Br J Ind Med* **45**: 843–844

Leiss JK and Savitz DA (1995) Home pesticide use and childhood cancer: a case–control study. *Am J Public Health* **85**: 249–252

Lowengart RA, Peters JM, Cicioni C, Buckley J, Bernstein L, Preston-Martin S and Rappaport E (1987) Childhood leukemia and parents' occupational and home exposures. *J Natl Cancer Inst* **79**: 39–46

McDowall ME (1985) *Occupational reproductive epidemiology. The use of routinely collected statistics in England and Wales 1980–82*. Studies on Medical and Population Subjects No. 50. HMSO: London

Magnani C, Pastore G, Luzzatto L and Terracini B (1990) Parental occupation and other environmental factors in the etiology of leukemias and non-Hodgkin's lymphomas in childhood: A case-control study. *Tumori* **76**: 413–419

Meinert R, Kaatsch P, Kaletsch U, Krummenauer F, Miesner A and Michaelis J (1996) Childhood leukaemia and exposure to pesticides: Results of a case-control study in northern Germany. *Eur J Cancer* **32A**: 1943–1948

Mulder YM, Drijver M and Kreis IA (1994) Case-control study on the association between a cluster of childhood haematopoietic malignancies and local environmental factors in Aalsmeer, The Netherlands. *J Epidemiol Comm Health* **48**: 161–165

Office of Population Censuses and Surveys (1970) *Classification of Occupations 1970*. HMSO: London

Office of Population Censuses and Surveys (1980) *Classification of Occupations 1980*. HMSO: London

Olshan AF, Breslow NE, Falletta JM, Grufferman S, Pendergrass T, Robison LL, Waskerwitz M, Woods WG, Vietti TJ and Hammond GD (1993) Risk factors for Wilms' tumor. Report from the National Wilms' Tumor Study. *Cancer* **72**: 938–944

Registrar General (1960) *Classification of Occupations 1960*. HMSO: London

Registrar General (1988) *Occupational Mortality 1979–80, 1982–83, England and Wales. Childhood Supplement*. HMSO: London

Schwartzbaum JA, George SL, Pratt CB and Davis B (1991) An exploratory study of environmental and medical factors potentially related to childhood cancer. *Med Pediatr Oncol* **19**: 115–121

Sharpe CR and Franco EL (1995) Etiology of Wilms' tumor. *Epidemiol Rev* **17**: 415–432

Sharpe CR, Franco EL, de Camargo B, Lopes LF, Barreto JH, Johnsson RR and Mauad MA (1995) Parental exposures to pesticides and risk of Wilms' tumor in Brazil. *Am J Epidemiol* **141**: 210–217

Stiller CA (1994) Population based survival rates for childhood cancer in Britain, 1980–91. *Br Med J* **309**: 1612–1616

Stiller CA and Bunch KJ (1990) Trends in survival for childhood cancer in Britain diagnosed 1971–85. *Br J Cancer* **62**: 806–815

Stiller CA, Allen MB and Eatock EM (1995) Childhood cancer in Britain: The National Registry of Childhood Tumours and incidence rates 1978–1987. *Eur J Cancer* **31**: 2028–2034

Stiller CA, McKinney PA, Bunch KJ, Bailey CC and Lewis IJ (1991) Childhood cancer and ethnic group in Britain: a United Kingdom Children's Cancer Study Group (UKCCSG) Study. *Br J Cancer* **64**: 543–548

Wilkins RJ (1988) Genomic imprinting and carcinogenesis. *Lancet* **1**: 329–331

Wilkins JR and Koutras RA (1988) Parental occupation and brain cancer in offspring: A mortality based case-control study. *Am J Ind Med* **14**: 299–318

Wilkins JR and Sinks TH (1984*a*) Occupational exposures among fathers of children with Wilms' tumor. *J Occup Med* **26**: 427–435

Wilkins JR and Sinks TH (1984*b*) Paternal occupation and Wilms' tumour in offspring. *J Epidemiol Comm Health* **38**: 7–11

World Health Organization (1957) *International Classification of Diseases. Seventh Edition. Manual of the International Statistical Classification of Diseases, Injuries and Causes of Death. Vol 1*. WHO: Geneva

World Health Organization (1967) *International Classification of Diseases. Eighth Edition. Manual of the International Statistical Classification of Diseases, Injuries and Causes of Death. Vol 1*. WHO: Geneva

World Health Organization (1977) *International Classification of Diseases. Ninth Edition. Manual of the International Statistical Classification of Diseases, Injuries and Causes of Death. Vol 1*. WHO: Geneva

Zack M, Cannon S, Loyd D, Heath CW, Falletta JM, Jones B, Housworth J and Crowley S (1980) Cancer in children of parents exposed to hydrocarbon related industries and occupations. *Am J Epidemiol* **111**: 329–336

© Cancer Research Campaign 1998